# Supplementary material for: Identification of photoperiod-induced specific miRNAs in the adrenal glands of Sunite sheep (Ovis aries)
Source: Front Vet Sci. 2022 Jul 22;9:888207. doi: 10.3389/fvets.2022.888207 (PMC9354845; doi:10.3389/fvets.2022.888207)
Supplement: Supplementary file 1 [file Data_Sheet_1.ZIP › Supplementary file/miRDeep2 file.zip!/miRDeep2 file/Know_Structure/LP42A1/oar-mir-200b.pdf]

miRBase precursor : oar-mir-200b
Total read count : 61
oar-miR-200b read count : 60
remaining reads : 1

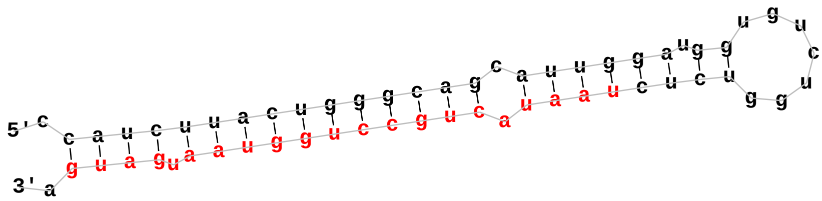

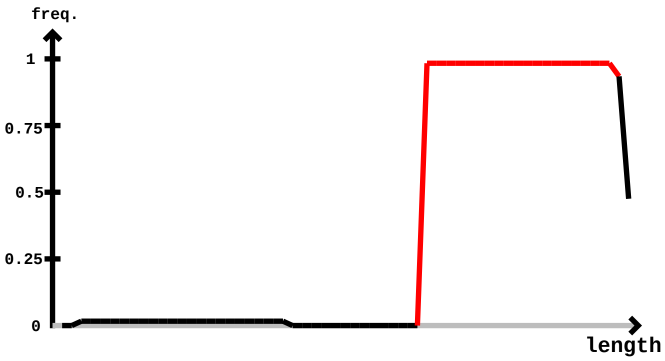


```
                                    oar-miR-200b
5'- ccaucuuacugggcagcauuggauggugucuggucucuaauacugccugguaaugauga   -3'    exp
    .(((((((((((((((.((((((.((.......)))))))).))))))))))).)))).    reads      mm         sample
    .caucuuacugggcagcauugga....................................    1          0          seq
    ....................................uaauacugccugguaaugau..    3          0          seq
    ....................................uaauacugccugguaaugaug.    28         0          seq
    ....................................uaauacugccugguaaugauga    29         0          seq
```
